# Acetabular Bone Cement Extension Leading to Bladder Obstruction: An Orthopedic Surgical Complication

**DOI:** 10.7759/cureus.68694

**Published:** 2024-09-05

**Authors:** Axel B Lichtenberg, Ali Z Ansari, Ghazwan Bahro, Muhammad Bilal, Sean Lief, Srihita Patibandla, Sahar Hafeez

**Affiliations:** 1 Department of Orthopedic Surgery, William Carey University College of Osteopathic Medicine, Hattiesburg, USA; 2 Department of Pathology, William Carey University College of Osteopathic Medicine, Hattiesburg, USA; 3 Department of Internal Medicine, Merit Health Wesley, Hattiesburg, USA; 4 Department of Internal Medicine, William Carey University College of Osteopathic Medicine, Hattiesburg, USA; 5 Department of Internal Medicine, Trinity Health Grand Rapids, Grand Rapids, USA

**Keywords:** bladder, bone cement, cemented total hip arthroplasty, foreign body extrusion, hematuria, hydronephrosis, osteoarthritis (oa), pelvis and acetabulum, recurrent urinary tract infections, shortness of breath (sob)

## Abstract

Polymethyl methacrylate, commonly known as bone cement, is widely used for implant fixation in orthopedic and trauma surgery due to its excellent adhesive properties and biocompatibility. However, complications such as bone cement extrusion, although rare, can lead to significant morbidity. We present the case of an 86-year-old Hispanic female who presented to the emergency department (ED) with tachycardia, hypertension, and respiratory distress. Her medical history included Parkinson's disease, hiatal hernia, osteoarthritis, colon cancer, and a complex post-hip fracture surgical history. Despite being bedridden, she had been previously in stable health. A computed tomography (CT) scan revealed a significant hiatal hernia, minimal remaining left lung tissue, a right lung nodule, hydronephrosis, and a large radiopaque mass in the right pelvis extending from the acetabular area. This radiopaque mass was later determined to be bone cement, with a portion extruding into the bladder. The patient was diagnosed with sepsis secondary to a urinary tract infection and hyponatremia; a urology consultation recommended a conservative approach to avoid potential antibiotic resistance. This case report highlights a rare complication of total hip arthroplasty involving bone cement extrusion into the bladder, which led to hydronephrosis and a urinary tract infection (UTI). Although such complications can be asymptomatic, they should be considered in patients with a history of arthroplasty.

## Introduction

Total hip arthroplasty is a widely performed and transformative orthopedic procedure used to treat hip joint disorders and degenerative conditions. This surgery involves replacing damaged or worn parts of the hip joint with artificial components made of metal, ceramic, or plastic, which helps restore joint function, alleviate pain, and improve mobility for many patients [1]. Despite its high success rate, the procedure carries several risks. The most common complications include dislocation (1-3%), thromboembolic events (1-2%), and implant loosening (0.2-10%) [2-4].

Foreign body extrusion into the pelvis following arthroplasty is a rare but significant complication. One such foreign body is bone cement, primarily composed of polymethylmethacrylate (PMMA), which is commonly used in orthopedic surgeries, including hip replacements, to secure implants and enhance joint stability [5]. PMMA is favored for its fast-setting properties, strength, and ability to bond well with surrounding bone structures. Additionally, it is radiopaque, which allows for visualization in imaging studies and is beneficial for monitoring its placement and detecting complications postoperatively [6].

However, the use of bone cement is not without risks. Rarely, bone cement may extend beyond the intended surgical site, leading to complications. This extrusion can occur due to high intramedullary pressures during the cementing process, which may cause the cement to leak into adjacent tissues [7]. Once extruded, the cement may initially remain stable or migrate over time, influenced by mechanical stress and biological factors such as tissue resorption or remodeling. Over time, this migration may allow the cement to gradually penetrate through surrounding soft tissues, eventually compromising nearby structures. In the context of hip arthroplasty, such migration poses a significant risk to adjacent pelvic organs, such as the urinary bladder, where cement infiltration may occur gradually, potentially facilitated by weakened or eroded intervening tissues. The case presented here involves the extrusion of bone cement from the acetabulum into the urinary bladder.

## Case presentation

An 86-year-old Hispanic female presented to the emergency department (ED) with complaints of shortness of breath. The patient communicated in Spanish, with her daughter serving as a translator. Her medical history was complex, including Parkinson's disease, hiatal hernia, osteoarthritis, and a history of colon cancer. She had been bedridden for several years due to complications from right hip fracture surgery, which included infection and subsequent osteoarthritis. Despite being bedridden and using diapers, she was able to feed herself and had been in generally good health until the sudden onset of her symptoms a few hours before admission. The patient's symptoms had been constant and worsening without any known aggravating factors. Further history revealed that she had undergone two surgical procedures on her right hip, the second of which, performed several years ago, involved the removal of the hip joint and some hardware. Additionally, she had a history of colon cancer resection and had lost her left lung, possibly due to a lung abscess, as suggested by her daughter.

Upon admission to the ED, the patient was afebrile, exhibited a heart rate of 109 beats per minute (bpm), a respiratory rate of 24 breaths per minute (bpm), a blood pressure of 180/135 mmHg, and an oxygen saturation of 95% on 2 liters of oxygen via nasal cannula. A review of systems highlighted significant shortness of breath, but the patient denied any associated symptoms such as fever, chills, chest pain, cough, urinary tract infection (UTI), flank pain, or abdominal pain. Physical examination revealed the patient lying in bed with decreased physical activity and signs of respiratory distress. The patient's hands appeared arthritic, with a decreased range of motion in both upper and lower extremities. She was alert and cooperative, with no other abnormalities noted on the physical exam.

The patient met the criteria for sepsis based on the presence of two systemic inflammatory response syndrome (SIRS) criteria: tachycardia with a heart rate of 109 bpm and tachypnea with a respiratory rate of 24 bpm, alongside clinical suspicion of infection. Despite the absence of fever or altered mental status, these findings, coupled with the suspicion of a UTI later supported by urinalysis, warranted this diagnosis. The urinalysis revealed numerous white blood cells, red blood cells, and elevated levels of blood and leukocyte esterase (Table 1). To confirm the source of infection and further assess her condition, a series of tests were conducted, including a complete blood count (CBC), comprehensive metabolic panel (CMP), arterial blood gas (ABG), lactate, estimated glomerular filtration rate (eGFR), cultures of blood and urine, and tests for influenza (flu) and COVID-19. ABG analysis indicated type 1 respiratory failure (Table 2), CBC revealed no abnormalities, and CMP showed hyponatremia (Table 3). All other tests conducted at this time returned normal results. Pending culture results prompted initial treatment with ceftriaxone, Lactated Ringer’s solution, intravenous (IV) fluids, and supplemental oxygen.

**Table 1:** Urinalysis demonstrated a high presence of white blood cells, red blood cells, and significantly elevated levels of blood and leukocyte esterase. HCG: Human chorionic gonadotropin

Components of Urinalysis	Value	Reference Range
Color	Yellow	Yellow
Clarity	Clear	Clear
Specific gravity	≥1.030	1.000-1.060
pH	6.4	5.0-9.0
Urobilinogen	0.9 mg/dL	0.1-1.8 mg/dL
Blood	Large	Negative
Glucose	0	0-15 mg/dL
Ketones	0.1 mg/dL	< 1 mg/dL
Protein	1.4 mg/dL	< 10 mg/dL
Nitrite	Negative	Negative
Leukocyte esterase	Positive	Negative
Epithelial cells	< 15 cells per HPF	< 15 cells per HPF
White blood cells	Too many to count	< 5 cells per HPF
Red blood cells	Too many to count	< 4 cells per HPF
β-HCG	Negative	Negative

**Table 2:** Arterial blood gas analysis indicated type 1 respiratory failure, characterized by decreased levels of PCO2, PO2, and oxygen saturation. PCO_2_: Partial pressure of carbon dioxide; PO_2_: Partial pressure of oxygen

Test	Observed Value	Reference Range
pH	7.44	7.35-7.45
pCO_2_	34 mmHg	35-45 mmHg
pO_2_	58 mmHg	75-100 mmHg
HCO_3_^-^	23 mEq/L	22-26 mEq/L
Oxygen saturation	89%	> 95%

**Table 3:** Laboratory findings from CBC and CMP showed hyponatremia as the only abnormality. CBC: Complete blood count; CMP: Comprehensive metabolic panel

Test	Observed Value	Reference Range
White blood cells	8.6 x 10^3^/μL	4.0-11.0 x 10^3^/μL
Red blood cells	4.3 x 10^6^/μL	4.0-5.0 x 10^6^/μL
Hemoglobin	14.7 g/dL	12.1-15.1 g/dL
Hematocrit	46%	42%-52%
Mean corpuscular hemoglobin	29 pg/cell	27-31 pg/cell
Mean corpuscular hemoglobin concentration	34 g/dL	33-36 g/dL
Mean corpuscular volume	91 fL	80–100 fL
Platelet count	353 x 10^9^/L	150-450 x 10^9^/L
Mean platelet volume	11 fL	8-12 fL
Red cell distribution width	13%	12%-15%
Sodium	131 mmol/L	135-147 mmol/L
Potassium	4.4 mmol/L	3.5-5.0 mmol/L
Chloride	98 mmol/L	96-106 mmol/L
Carbon dioxide	26 mmol/L	23-29 mmol/L
Blood urea nitrogen	5.5 mmol/L	2.1-8.5 mmol/L
Creatinine	1.0 mg/dL	0.7-1.3 mg/dL
Glucose	91 mg/dL	70-100 mg/dL
Calcium	8.8 mg/dL	8.5-10.2 mg/dL
Albumin	4.7 g/dL	3.5-5.5 g/dL
Alkaline phosphatase	123 U/L	44-147 U/L
Alanine aminotransferase	43 U/L	7-56 U/L
Aspartate aminotransferase	31 U/L	5-40 U/L
Total bilirubin	0.8 mg/dL	0.3-1.0 mg/dL
Total protein	6.8 g/dL	6.0-8.3 g/dL
Globulin	3.1 g/dL	2.0-3.5 g/dL
Lipase	113 U/L	10-140 U/L

**Figure 1:**
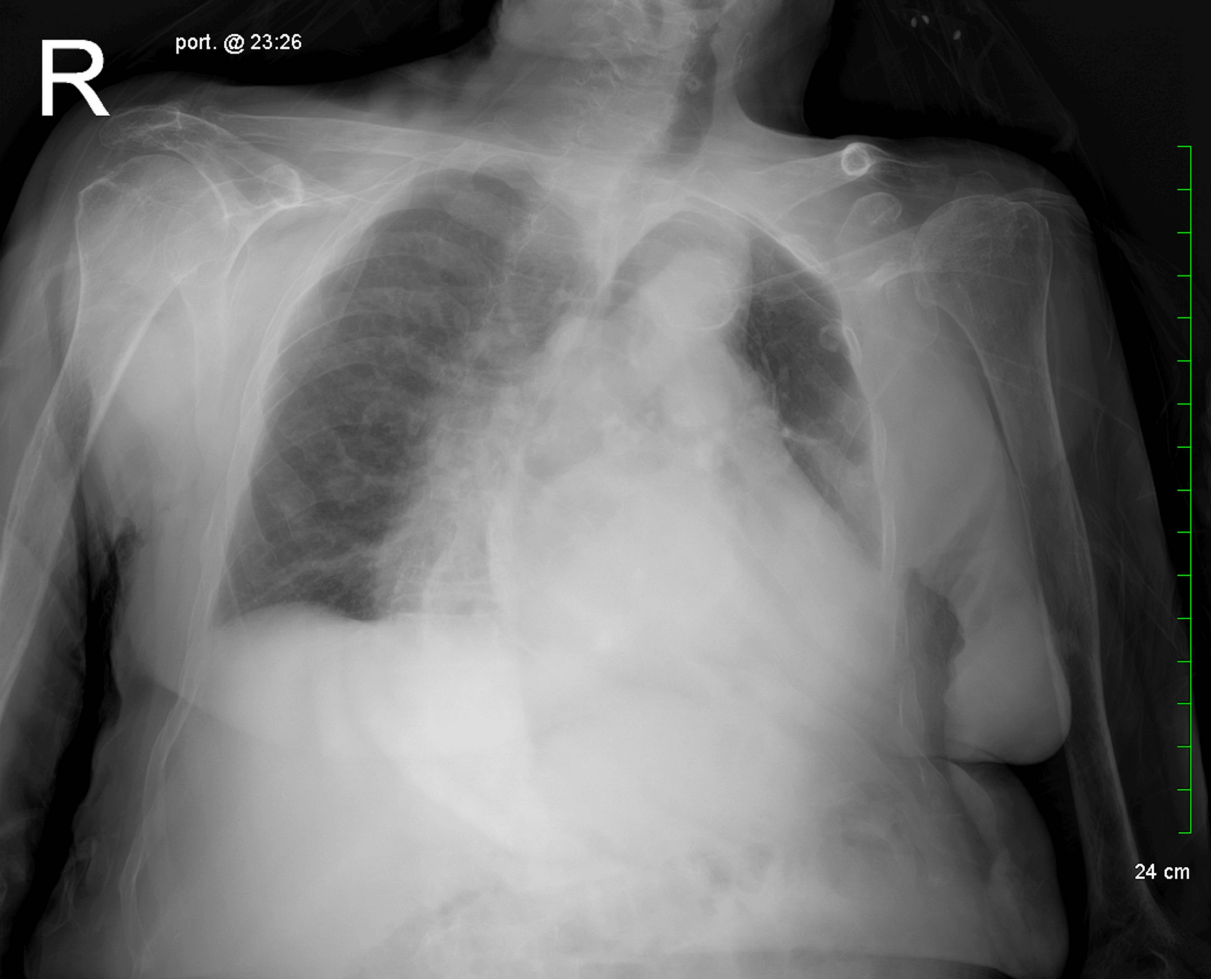
Anteroposterior view X-ray of the chest with the patient rotated to the left. The X-ray shows old fractures, left deviation of the mediastinum, possibly in part due to the resected lung tissue, and cardiomegaly with an unknown density posterior to the heart.

Initial imaging with a portable chest X-ray was performed (Figure 1) to evaluate the cause of her shortness of breath. The X-ray revealed diffuse osteopenia with old bilateral rib fractures, an old fracture of the left clavicle, and signs of cardiomegaly. Additionally, there was leftward deviation of the mediastinum, atherosclerotic calcifications of the aorta, and a large, undetermined density posterior to the heart.

**Figure 2:**
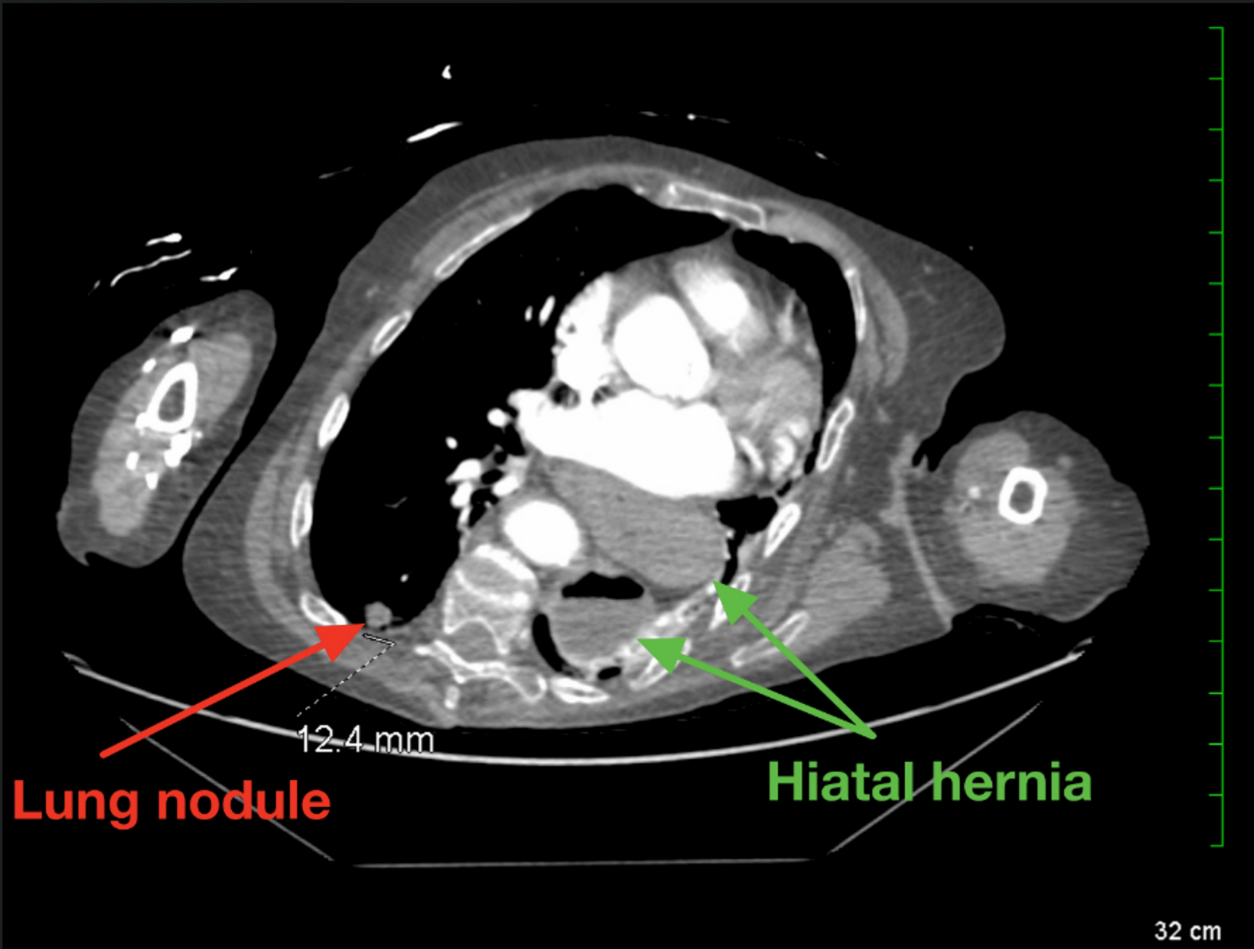
CT angiography of the chest showing a right lung nodule (red arrow) and a hiatal hernia (green arrows). CT: Computed tomography

**Figure 3:**
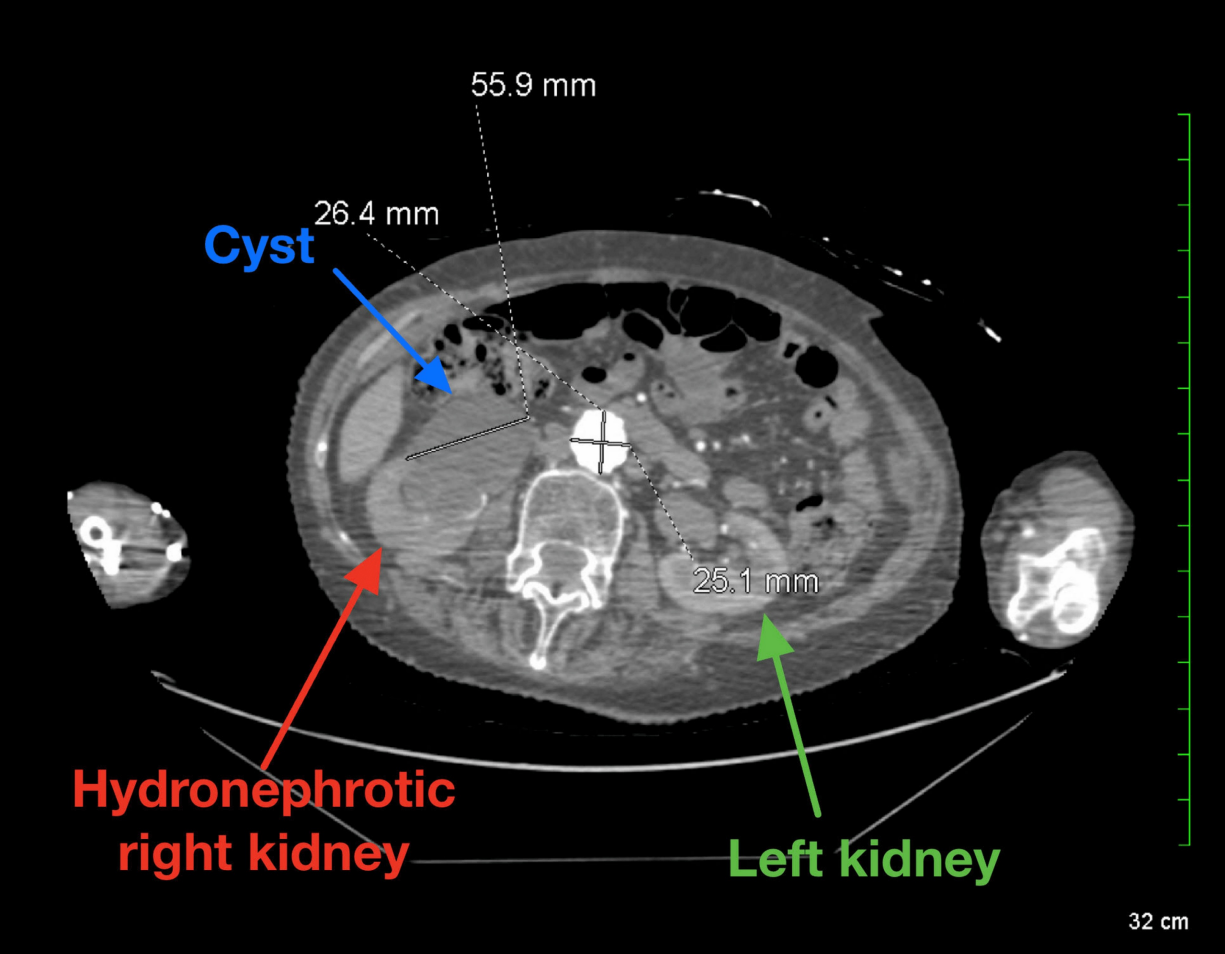
CT angiography of the abdominal region demonstrating right kidney hydronephrosis (red arrow) and a cyst (blue arrow). CT: Computed tomography

**Figure 4:**
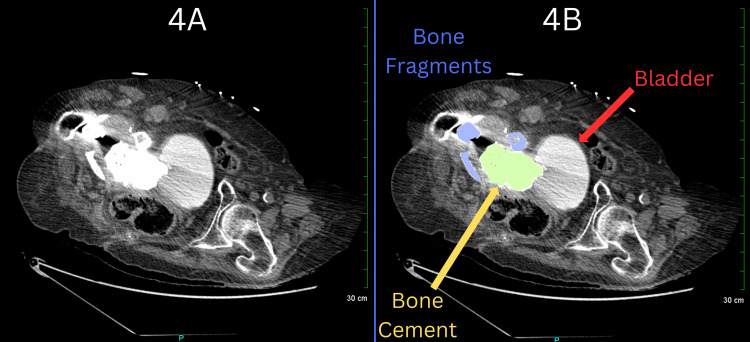
CT angiography of the pelvic region. Panel 4A displays the unlabeled image, while Panel 4B presents the labeled image for anatomical reference. The bone cement is highlighted in yellow, protruding into the bladder as indicated by the red arrow. Bone fragments, marked in blue, are visible on the right side of the body. CT: Computed tomography

The subsequent computed tomography (CT) scan revealed a large hiatal hernia projecting to the left of the midline, with minimal remaining left lung tissue and a 12 mm nodule in the right lower lung, highly suspicious for malignancy (Figure 2). The hiatal hernia was associated with compressive atelectasis in the left lower lobe. Additionally, the scan showed moderately severe right-sided hydronephrosis and a 5.6 cm cyst along the anterior aspect of the right kidney (Figure 3). In the pelvic region, a 4 to 5-cm radiopaque mass, suggestive of bone cement, was identified as contiguous with the right side of the bladder and potentially protruding into the bladder (Figure 4). The scan also revealed dilation of the ureter down to the level of the obstruction at the bladder and multiple thyroid nodules.

The differential diagnosis at this time included sepsis secondary to a urinary tract infection, hyponatremia potentially attributable to paraneoplastic syndrome or dehydration, a large hiatal hernia, and a pulmonary nodule that could be metastatic from the patient’s previous colorectal cancer. Given the complexity of the patient's presentation, initial management focused on addressing these concerns while ensuring her ongoing carbidopa/levodopa regimen for Parkinson's disease was continued. Further diagnostic clarity was anticipated from the pending culture results.

Given the patient's complex medical history and hydronephrosis, urology was consulted the following day. The urologist reviewed the CT scans and confirmed our initial findings: a large, dense abnormality extending from the acetabulum into the pelvis, consistent with bone cement, with a portion protruding into the bladder. He confirmed that the bone cement extrusion was indeed the likely cause of the patient's symptoms. Given these findings and the patient’s stable renal function, the urologist recommended a conservative approach rather than intravenous antibiotics due to concerns about antibiotic resistance. Additional imaging techniques, such as cystoscopy and retrograde pyelograms, were considered to further evaluate the extent of the extrusion, along with the option of a radionuclide renal scan with split renal function and a furosemide washout to assess the right kidney's functionality. However, these procedures were ultimately deemed unnecessary due to the potential risk of introducing infections that could necessitate permanent percutaneous nephrostomies.

The patient remained stable and was managed conservatively for the remainder of her stay in the ED. Later in the day, she reported feeling improved and experiencing no pain. Given her stable clinical status, absence of pain, and no further complications, she was discharged home with detailed follow-up instructions. She was advised to see a urologist within two weeks for further evaluation and management of the bone cement extrusion into the bladder. Additionally, she was instructed to follow up with her primary care physician (PCP) for further assessment of the thyroid nodules and the pulmonary nodule identified on her chest CT.

## Discussion

Studies have shown that periacetabular bone cement extrusion occurs in 25-44% of total hip arthroplasty cases [8,9]. However, the specific complication of bone cement extrusion into the bladder is exceedingly rare, with only a few cases documented in the literature [10-18]. A thorough review of case reports highlights various instances of cement migration leading to significant complications. For example, one study reported delayed ureteric injury resulting from thermal damage during hip replacement surgery [15]. Another study documented bladder and ureteral displacement as a complication of total hip arthroplasty [16]. Additional cases include a report of ureteric obstruction following hip replacement and a vesico-acetabular cutaneous fistula as a delayed complication of hip surgery [17,18]. These cases demonstrate the range of genitourinary complications that can arise from orthopedic procedures.

A particularly notable case involved a 70-year-old female who presented with a mass in the bladder wall 21 months after undergoing a right hip joint replacement. The patient required partial resection of the bladder to remove the extruded bone cement and subsequently experienced an uneventful recovery postoperatively [11]. This case, along with the others reviewed, emphasizes the importance of considering bone cement extrusion as a potential cause in patients presenting with persistent urinary tract symptoms following hip surgery.

The differential diagnosis for our patient's condition included the possibility of sepsis secondary to a UTI, supported by infection-related symptoms and evidence of hydronephrosis. The diagnosis of sepsis was complicated by the overlap of symptoms with other serious conditions, including potential complications from the patient’s orthopedic history. The extrusion of bone cement into the bladder added another layer of complexity, potentially exacerbating or mimicking symptoms of infection. The management strategy, including the decision to use conservative treatment, reflects the need to carefully evaluate and address all possible contributing factors to avoid worsening the patient's condition and to optimize outcomes.

Treatment strategies for bone cement extrusion can vary significantly depending on the severity of the condition and the patient's symptoms. In our patient's case, conservative management was selected, which involved treating the UTI and closely monitoring renal function. However, the literature also describes more invasive approaches for symptomatic patients experiencing severe complications, such as percutaneous endoscopic procedures or open surgical removal of the extruded bone cement [19,20].

For future patients presenting with similar complications, a comprehensive risk-benefit analysis should guide the treatment strategy. Conservative management may be appropriate for asymptomatic patients with stable renal function, while symptomatic patients might require more aggressive interventions. Additionally, postoperative imaging and long-term follow-up are crucial for the early detection and management of such rare complications. A limitation of this case report is the lack of follow-up data, as the patient was discharged from the emergency department with instructions to follow up with her primary care physician and did not report back to our facility. This limits our ability to assess the long-term outcomes over time. Future research should aim to develop standardized protocols for managing bone cement extrusion and identify predictive factors for these complications. Studies investigating minimally invasive techniques for the safe and effective removal of extruded bone cement could lead to significant improvements in patient care.

## Conclusions

This case report highlights an exceptionally rare complication of total hip arthroplasty involving the extrusion of bone cement into the bladder, which resulted in hydronephrosis and a UTI. Although the patient’s complex medical history and the decision to pursue conservative treatment were critical factors in this case, there is a broader need for standardized protocols to explore minimally invasive approaches for managing such complications. Future research should focus on developing clearer guidelines and identifying predictive factors to improve care and outcomes for patients undergoing orthopedic surgeries involving bone cement.
